# A systematic review: facial, dental and orthodontic findings and orofacial diagnostics in patients with FASD

**DOI:** 10.3389/fped.2023.1169570

**Published:** 2023-06-08

**Authors:** Moritz Blanck-Lubarsch, Dieter Dirksen, Reinhold Feldmann, Ariane Hohoff

**Affiliations:** ^1^ Department of Orthodontics, University of Münster, Münster, Germany; ^2^ Department of Prosthodontics and Biomaterials, University of Münster, Münster, Germany; ^3^ Department of Pediatrics, University of Münster, Münster, Germany

**Keywords:** review, craniofacial anomalies, orofacial disorder, diagnostic methods, fetal alcohol syndrome (FAS)

## Abstract

**Background:**

The fetal alcohol spectrum disorder is a group of developmental disorders caused by maternal alcohol consumption. Patients with fetal alcohol syndrome show abnormal orofacial features. This review presents an overview over the facial, oral, dental or orthodontic findings and diagnostic tools concerning these features.

**Methods:**

For this systematic review Cochrane, Medline and Embase databases were considered and the review was performed according to the PRISMA checklist. Two independent reviewers evaluated all studies and recorded results in a summary of findings table. Risk of bias was analyzed via Quadas-2 checklist.

**Results:**

61 studies were eligible for inclusion. All included studies were clinical studies. Methods and results of the studies were not comparable, guidelines or methods for the detection of FASD varied across studies. Facial features most often measured or found as distinguishing parameter were: palpebral fissure length, interpupillary or innercanthal distance, philtrum, upper lip, midfacial hypoplasia or head circumference.

**Conclusions:**

This review shows that to date a multitude of heterogeneous guidelines exists for the diagnosis of FASD. Uniform, objective diagnostic criteria and parameters for the orofacial region in FASD diagnosis are needed. A bio database with values and parameters for different ethnicities and age groups should be made available for diagnosis.

## 1. Introduction

### 1.1. What is FASD?

FASD is a group of developmental disorders, which result from maternal alcohol exposure. Symptoms comprise growth deficiencies, aberrant facial features and damage or dysfunction of the central nervous system (1–3). Individuals with FASD suffer from lifetime consequences, even more if the underlying disorder remains undiagnosed.

Different severity grades of the disorder are summarized by the term fetal alcohol spectrum disorder (FASD). The most severe form of FASD is the fetal alcohol syndrome (FAS), less severe forms are categorized as partial fetal alcohol syndrome (pFAS), alcohol-related birth defects (ARBD) or alcohol-related neurodevelopmental disorder (ARND) (4–6).

### 1.2. Prevalence of FASD, FAS, pFAS, ARND, ARBD

Unfortunately, the estimated worldwide prevalence of the fetal alcohol spectrum disorder is still very high (0,77%, Lange et al., 2017), although it should be common knowledge that any amount of alcohol consumption during pregnancy is harmful to the embryo. FASD shows regional differences ranging from up to 5% in first grade school children in the United States (7), 2%–3% in Canadian school children (8), 1.98% in Europe to 0.01% in the eastern Mediterranean region (9). According to a Canadian study by Popova et al. the estimated prevalence for school children aged 7–9 years is 1.2 per 1,000 for FAS, 2.0 per 1,000 for pFAS and 15 per 1,000 for ARND (8). There is no recent prevalence for ARBD in literature, a study from 1988 by Warren et al. mentions a high prevalence of 417 per 1,000 (10).

### 1.3. Diagnosis of FASD

FASD is a developmental disorder, which is, because of its complexity and variable severity, challenging to detect. Since Lemoine first described the distinctive features and established the disorder FAS, a great number of diagnostic systems have been developed (11).

The current variability of existing diagnostic methods concerning FASD impedes comparability and reproducibility (12, 13).

There seem to be four guidelines across the world, which are most commonly used but which contain differences in certain diagnostic parameters:

- The four-digit code by Astley et al.—not for the terms ARND/ARBD (14)
- The revised IOM by Hoyme (5)
- The Canadian guideline by Chudley (6)
- The CDC code—mainly for FAS, not for partial FAS/ARND/ARBD (15)

Most diagnostic guidelines for FASD include **four parameters: growth deficiency, facial phenotype, central nervous system (CNS) damage or dysfunction as well as gestational exposure to alcohol** (5, 16). The most uncertain parameter is in many cases the exposure to alcohol since many children with FASD live in foster care or mothers might not tell the truth about alcohol consumption during pregnancy (17).

**Growth retardation** is measured according to percentile curves and for the guidelines mentioned above, measurements should be below the 10th percentile to be considered relevant for FAS diagnosis. In the case of partial FAS this measurement is only required for the IOM guideline and not necessary for diagnosis in the other guidelines. For ARBD and ARND growth retardation is not part of the diagnosis (5, 6, 14, 15).

**CNS** involvement is measured differently in the mentioned guidelines. For FAS the four-digit code requires a head circumference, which is smaller by more than two standard deviations below the 2.5th percentile, whereas the revised IOM and the CDC require only measurements below the 10th percentile for FAS diagnosis. The Canadian guideline does not measure according to percentile curve but requires more than three impairments in the CNS domains, for example hard and soft neurologic signs, brain structure, cognition, academic achievement, memory. For partial FAS CNS involvement is the same as for FAS in the four-digit code and the Canadian guideline, not applicable for the CDC guideline and for the revised IOM head circumference should be below the 10th percentile or behavioral and cognitive abnormalities should be present. ARND requires neurobehavioral impairment for the IOM and for the Canadian guideline evidence of CNS neurodevelopmental abnormalities and/or evidence of a complex pattern of behavior or cognitive abnormalities. ARBD requires one or more specific cardiac, skeletal, renal, eye or ear malformation for both the IOM and the Canadian guideline (5, 6, 14, 15).

In all four diagnostic guidelines **alcohol exposure** must not necessarily be confirmed for FAS. For partial FAS the four-digit code and the Canadian guideline require confirmed alcohol exposure, whereas the revised IOM does not require confirmed alcohol exposure for diagnosis of partial FAS. For ARND and ARBD confirmed alcohol exposure is required in the revised IOM and Canadian Guideline (5, 6, 14, 15).

For the parameter **facial phenotype** a commonly used tool is the lip-philtrum guide by Astley and Clarren (18). Facial characteristics used in the diagnostic guidelines comprise short palpebral fissures, thin vermillion borders and a smooth philtrum. Short palpebral fissure lengths are measured using a ruler or are measured via computer using the FAS facial photographic analysis software by Astley et al. The smooth philtrum and thin vermillion border are diagnosed comparing the patient's facial features to pictures on the lip-philtrum guide by Astley and Clarren and then ranking them to a certain grade of the feature (14, 18, 19).

All currently available diagnostic guidelines for the detection of facial FASD-parameters are in parts based on subjective evaluation. Therefore, this requires a professional dysmorphologist with experience in this field and is otherwise another factor for possible bias in the FASD diagnosis.

### 1.4. Aim of the review

Newer methods for the evaluation of facial parameters have investigated shape analysis, morphometrics, 3D-facial scanning and stereophotogrammetry with promising results for objectifying facial analysis in the future (20–35).

To improve the diagnostic process in patients with FASD it seems important to find facial characteristics for reproducible and objective measurements.

The aim of this review was to analyze, which facial, oral, orthodontic and dental parameters for the detection of FASD have been found so far and what kind of different diagnostic methods have been described for the diagnosis of FASD.

## 2. Materials and methods

### 2.1. Protocol and eligibility criteria

This review was performed according to the PRISMA guidelines. It was confined to articles in English, French or German that were published until January 2022.

Studies were considered eligible for inclusion if facial or oral structures in patients with FASD were investigated or if diagnostic methods for abnormal facial or oral structures in patients with FASD were described or tested.

Exclusion criteria were: Animal studies, scientific papers in which exposure of the study subjects to alcohol during pregnancy was not clear and studies concerning exclusively brain structures or ophthalmologic findings other than external ophthalmologic findings such as palpebral fissure length, innercanthal or interpupillary distance.

### 2.2. Search strategy

PICO Question was defined (Table 1).

**Table 1:** Showing the PICO question.

PICOs	
Participants	Children with FASD, FAS, pFAS, ARND, ARBD
Intervention or exposure	Facial, dental or orthodontic findings or orofacial diagnostic methods
Comparison or control	Patients without prenatal alcohol exposure
Outcome measure(s)	Anatomic measurements of length, width or depth and/or dental/orthodontic score(s), diagnostic method(s)
Type of Studies included	Clinical studies

Cochrane, Medline and Embase databases were considered. The search strategy was: [fetal alcohol syndrome OR “fetal alcohol spectrum disorders” (MESH)] AND (“Face” [MESH] OR “Mouth” [MESH] OR “Oral Health” [MESH] OR “orthodontics” [MESH] OR “dental” OR “Head” [MESH] OR “palpebral fissure length” OR “diagnostic” OR “diagnose”).

### 2.3. Study selection

All studies were screened for eligibility criteria.

### 2.4. Data extraction

All studies were screened by two independent reviewers MBL, AH in a blinded manner and classified into “eligible” or “not eligible” for this review. All “eligible” studies were read in full-text by the two reviewers and evaluated according to the Quadas-2 checklist. Data concerning facial features in patients with FASD and FASD diagnostic methods for the orofacial region were extracted independently by the two reviewers and recorded in a summary of findings table.

After reading all eligible studies full-text the two reviewers discussed their results and re-read those studies for which results differed in order to find a consensus.

### 2.5. Data items

The following outcomes were extracted:

• FASD or subgroup (FAS, pFAS, ARND, ARBD) diagnosis
• Control group
• Ethnicity
• Age
• Results, metric facial measurements or diagnostic scores of
  • Head circumference (OFC = occipital frontal circumference)
  • Palpebral fissure length (PFL), distance of inner and other canthi of the eye
  • Innercanthal distance (ICD), distance between the inner canthi of right and left eye
  • Interpupillary distance (IPD), distance between the centers of the pupils or right and left eye
  • Philtrum anomalies in sagittal, vertical and transversal dimension
  • **d**ecayed **m**issing **f**illed **t**eeth index for deciduous teeth (dmft) and permanent teeth (DMFT), the sum of the number of decayed, missing due to caries and filled teeth
  • DDE index (classification of **d**evelopmental **d**efects of **e**namel),
  • facial or oral features associated with FASD
• applied/(new, for example 3D or computer aided) diagnostic methods for the orofacial region
• aim of the study

### 2.6. Synthesis

Synthesis of data was not possible due to inconsistent measuring methods and inhomogeneity across the studies.

### 2.7. Effect measures

Effect measures were not possible due to inhomogeneity across the studies.

### 2.8. Bias/quality assessment

To minimize bias, two reviewers independently selected eligible studies for this review according to afore defined criteria and did independent full-text reading and data extraction of all included studies.

The quality of the studies was assessed using the Quadas-2 checklist. The Quadas-2 checklist is supposed to evaluate the quality of diagnostic accuracy studies.

Possible bias for the synthesis of data was recorded if:

• the diagnostic guideline for FASD was unclear or not stated.
• the diagnosis FAS, FASD, pFAS, ARND or ARBD was not clearly defined.
• ethnicity was not described or unclear.
• the method was not fully described or unclear.
• the participants' ages did not match or were not clearly stated.

## 3. Results

### 3.1. Study selection (flow of studies)

**Figure 1:**
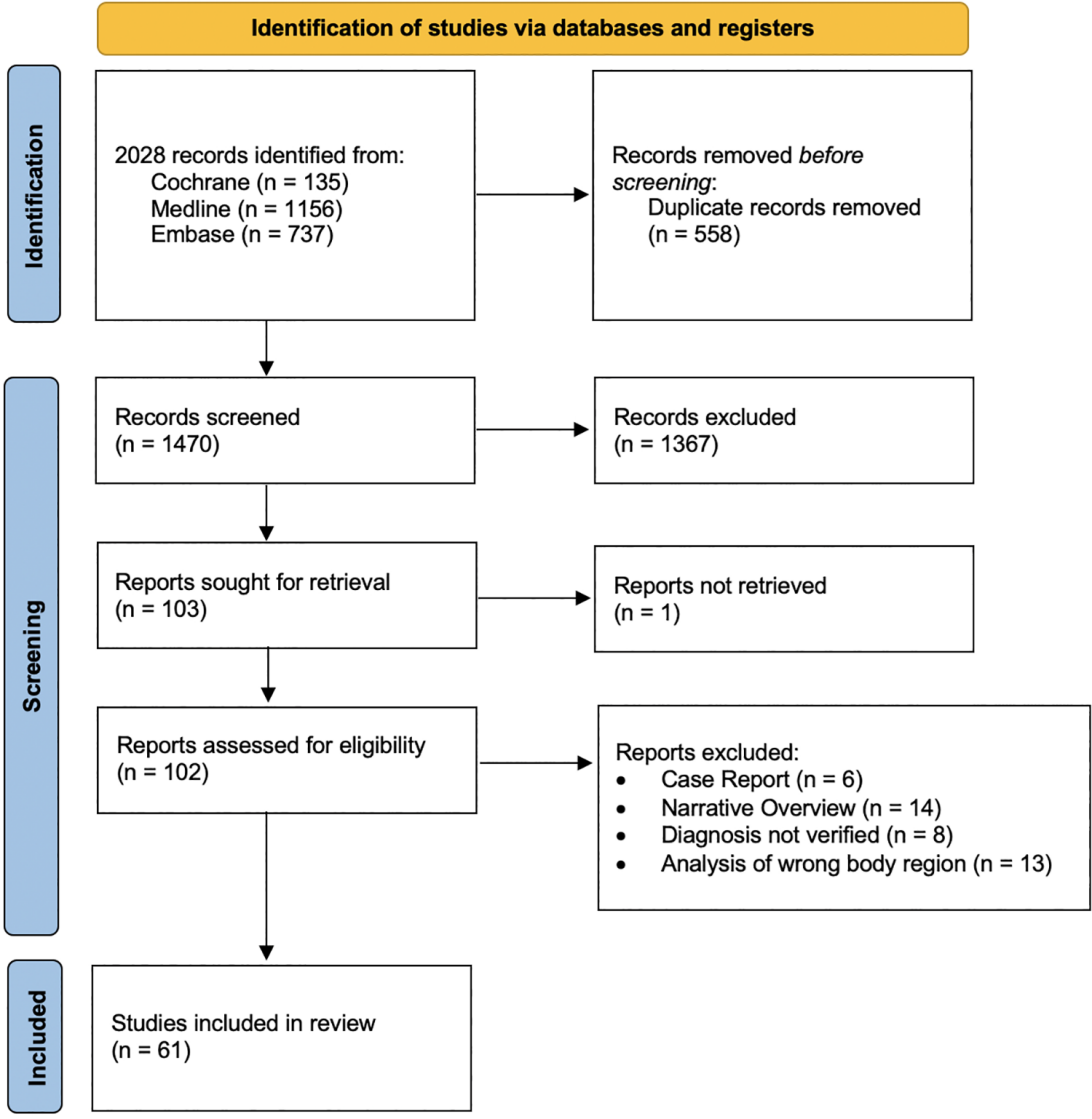
PRISMA flowchart. The search strategy resulted in 1,470 studies. A total of 1,409 studies were excluded. 61 studies were included in the review (84).

The search strategy resulted in a total of 1,470 studies (Figure 1).

### 3.2. Study selection (excluded studies)

1,409 studies were excluded (Figure 1).

### 3.3. Study characteristics

**Figure 2:**
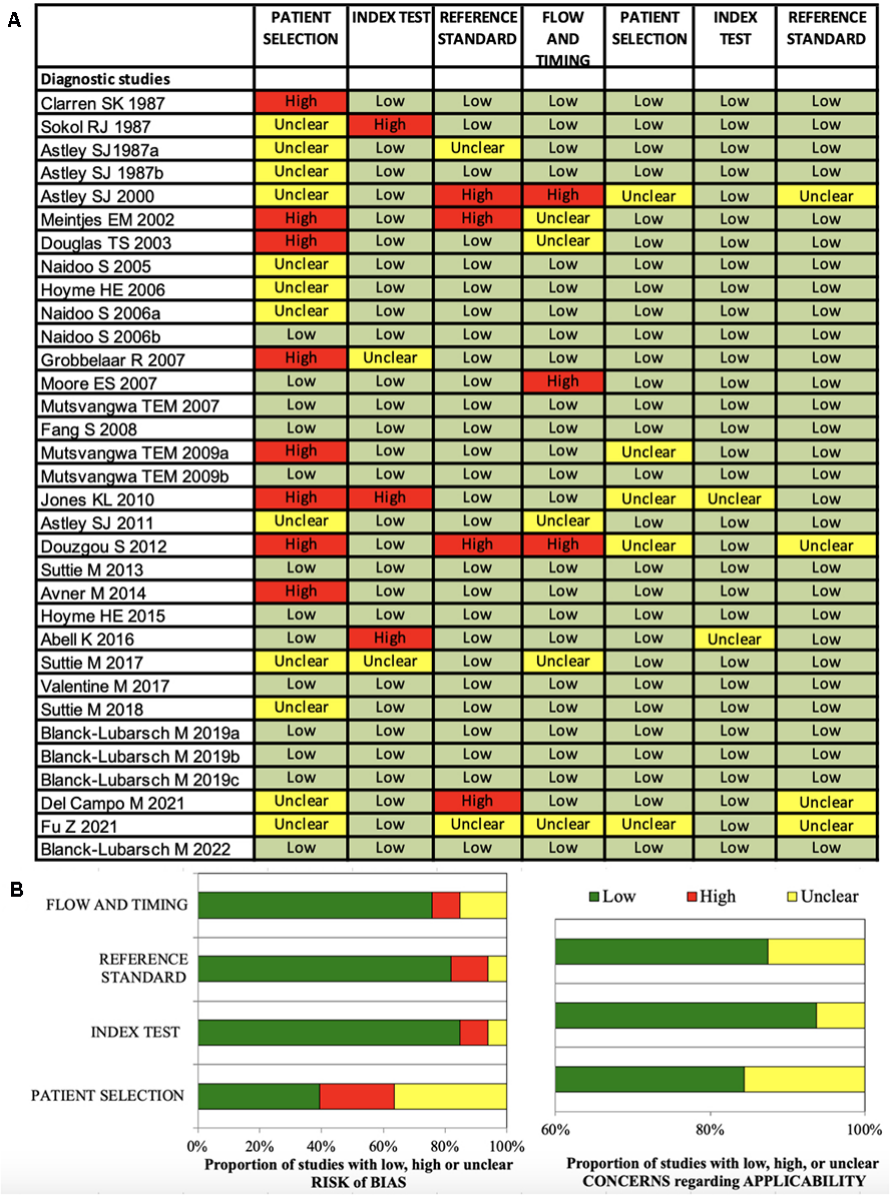
(**A,B**) Quadas-2 checklist for diagnostic studies. The evaluation showed low risk of bias concerning flow and timing, reference standard and index test. Risk of bias in patient selection was low for about 39% of the studies (85).

**Figure 3:**
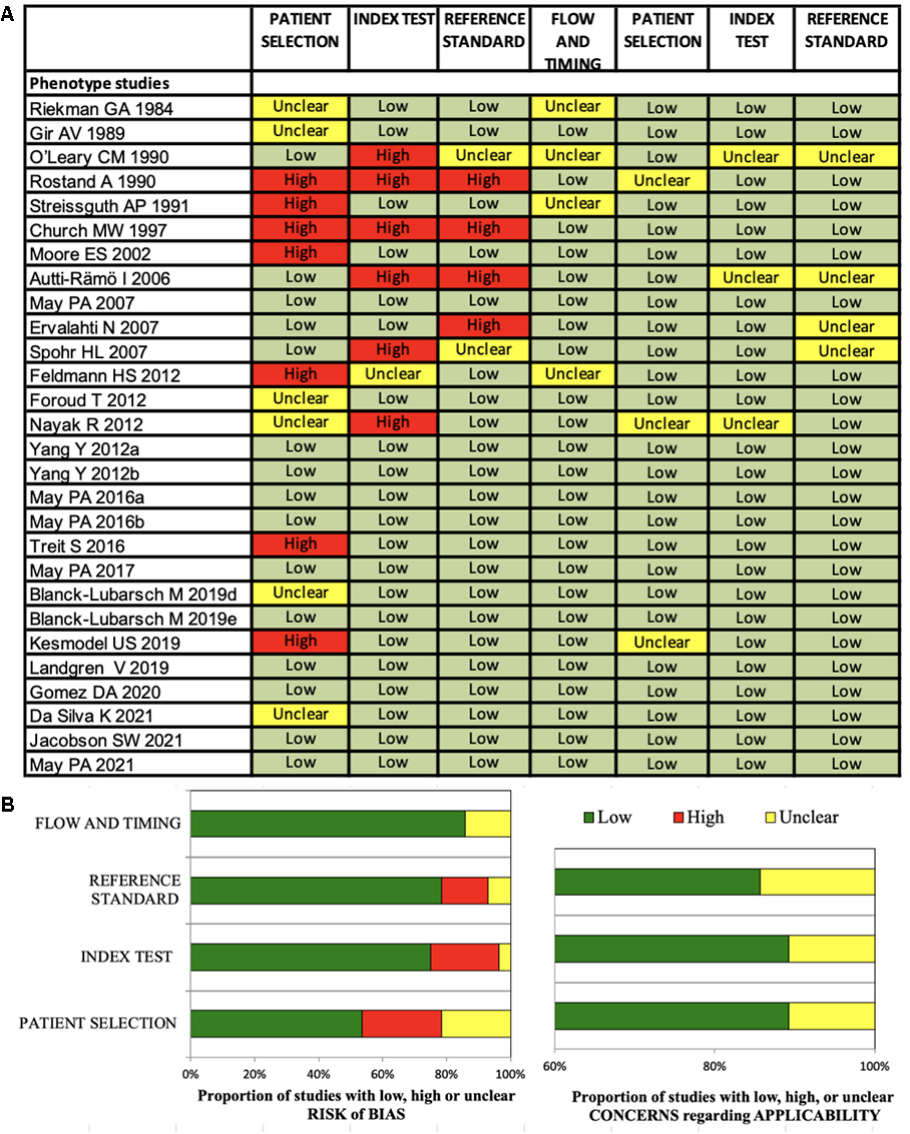
(**A,B**) Quadas-2 checklist for phenotype studies. The evaluation showed low risk of bias concerning flow and timing, reference standard and index test. Risk of bias in patient selection was low for about 54% of the studies (85).

All 61 included studies were clinical studies: 33 studies investigated diagnostic methods (Figure 2A) and 28 studies investigated the FASD phenotype (Figure 3A).

### 3.4. Risk of bias in studies

The Quadas-2 evaluation showed low risk of bias concerning flow and timing (diagnostic studies 75%, phenotype studies 86%), reference standard (diagnostic studies 81%, phenotype studies 79%) and index test (diagnostic studies 84%, phenotype studies 75%) (Figures 2B, 3B). Risk of bias in patient selection was low for 39% of the diagnostic studies and for 54% of the phenotype studies. This was for example due to unclear or missing clarification of ethnicities or unclear verification of the FASD diagnosis.

None of the studies was rated with high concerns regarding applicability.

### 3.5. Results of individual studies

The results of the individual studies are displayed in Table 2.

### 3.6. Guidelines used for FASD diagnosis

The guidelines for FASD diagnosis used within the studies were the IOM (5 studies), the revised IOM (14 studies), the 4-digit diagnostic guide (4 studies), the German guideline (5 studies) and the Canadian guideline (1 study) (5, 6, 18, 36, 37).

**Table 2:** Showing the complete list of included studies and investigated parameters.

Author	Year	Ethnicity (nomenclature as mentioned in the respective study)	Population (Age)	Population (*n*=)	Control (*n*=)	Guideline used	Diagnosis FASD/FAS/PAE	Measurements	Results	Research Aim
Clarren SK	1987	White (15), Black (5), American Indian (1)	7.00	21 (2 FAS)	21	Alcohol consumption mother	FAS/probable FAE/possible FAE	2D photographs; landmarks face lateral/frontal	Short palpebral fissure length (PFL), long and flat midface, retrusive mandible	Expert diagnostics via photographs/facial morphometric analysis via landmarks
Sokol RJ	1991	Unclear	Newborn	21/25?	76	Alcohol consumption mother	FAS	Frontal/lateral Polaroid snapshots; landmarks face lateral/frontal	Short PFL, scoping out of the nasal bridge, thin vermilion	Facial features in neonates
Astley SJ	1995	African American; American Indian; Alaskan Native; Asian; other	5.20	194/27 FAS	no	Trained dysmorphologist, Gestalt diagnosis	FAS/pFAS	Expert evaluation	Screening tool parameters: PFL, philtrum smoothness, upper lip thinness, minimal influence by gender, race and age up to 10	Development of effective screening tool
Astley SJ	1996	White, Black, American Indian, Alaskan Native, Asian, Hispanic	6.50 (0−27)	42	84	expert	FAS	Frontal photographs	Parameters: Reduced PFL and innercanthal distance ratio, smooth philtrum, thin upper lip; unaffected by gender, race and age	Development of FAS diagnosis via photographic screening tool
Astley SJ	2000	Caucasian 57.5%; African American 9%; Native American/Alaskan 14.1%; other 19.4%	Birth to 51 mth; mean: 10.1 (+/- 7)	454 (FAS 69, AFAS 41, PFAE 344)	no	Gestalt diagnosis	FAS/AFAS/PFAE	Gestalt diagnosis vs. 4-digit diagnostic code	Classification with Gestalt and 4-digit diagnostic code is variable. Inter- and intrarater reliability of the 4-digit code (for 20/16 patients) was high	To describe and present preliminary assessments of the accuracy, precision and power of the 4-digit diagnostic code so that others can consider and evaluate its use
Meintjes EM	2002	Not mentioned	First grade children	0	44	Two dysmorphologists	Children with growth retardation	3D photographs vs. handheld ruler measurement, PFL, ICD and IPD	PFL is suitable for 3D analysis; 3D measurements for PFL, ICD and IPD are highly repeatable ICD and IPD	Evaluation of measurements on 3D photographs vs. measurements via handheld ruler
Douglas TS	2003	South Africa	6−7	0	46	FAS screening	Unclear/FAS screening	3D photographs; manual vs. automatic extraction of eye features	Values for PFL and IPD were within 1 mm, whereas ICD and OCD showed ICD and IPD greater differences	Comparison between the automatic vs. manual extraction of eye features in 3D photographs for PFL, IPD, ICD and OCD ICD and IPD
Naidoo S	2005	South Africa	8.90	90	90	Not mentioned	FAS	decayed missing filled teeth (dmft), DMFT, enamel opacities, plaque score, gingival bleeding	dmft FAS: 1.79 vs. control: 1.63; DMFT FAS 0.77 vs. control 0.94 anthropometry; 42% of the FAS sample manifested statistically significant growth retardation, more plaque and gingival bleeding but not significant; no difference in enamel opacities; crowded incisors, maxillary overjet and openbite	Anthropometric measures and oral health
Hoyme HE	2006	Native American; South African	Not mentioned	164	Yes (matched control children)	Institute of Medicine (IOM)	FAS/pFAS/ARND/ARBD	PFL, morphologic features of the philtral ridges and the upper lip	Revised IOM criteria	To present specific clarifications of the 1996 IOM criteria for the diagnosis of FASD, to facilitate their practical application in clinical pediatric practice
Naidoo S	2006^a^	Not mentioned	FAS: 8.90 Control: 9.1	90	90	IOM	FAS	Cephalometric analysis, cranial base, midface, mandible, soft tissue profile	Vertically and horizontally underdeveloped maxilla, longface syndrome with large gonial angle and a short ramus in relation to total face height; tendency anterior open bite	Cephalometric findings in patients with FAS
Naidoo S	2006^b^	coloured	FAS: 8.95 Control: 9.04	90	90	IOM	FAS	Panoramic radiographs, handwrist radiograph, dental maturaty, skeletal age, chronological age	Dental delay score was significantly lower for male children with FAS compared with control	Analysis of dental vs. skeletal age in patients with FAS
Grobbelaar R	2007	South African	6.50	0	48	Not mentioned	No FASD diagnosis	Eye measurements, upper lip circularity	Introduction of an approximation to the upper lip circularity, which replaces manual outlining of the lip on a digital image with more time-efficient selection of four points on the image; automated point selection is not suitable for eye measurements	Area based matching, stereophotogrammetry, manually vs. automatically selected reference points
Moore ES	2007	North American Caucasian, Finnish Caucasian, African American, Cape Coloured	NAC: FAS 10.9, control 11.1; AA: FAS 11.3, control 6.5; FC: FAS 13.1, control 13.8; CC FAS 5.6, control 5.3	124	152	Revised IOM	FAS	4 different populations (North American Caucasian, Finnish Caucasian, African American, Cape Coloured); 6 scans per participant, 3D images, customized software, 20 landmarks, 16 measurements	FAS can be effectively discriminated from controls in Caucasians and African admixture. The facial feature, which most effectively discriminates FAS and controls differed across the populations	The purpose of the study was to test whether computerized anthropometry can distinguish patients with FAS from controls across a wide age range as well as across ethnically disparate study populations
Mutsvangwa TEM	2007	South African	FAS 7.11; controls 6.56	14	20	Expert/3 independent dysmorphologists	FAS	Procrustes, principal component analysis, face shape analysis, facial landmarks	No difference between control and FAS when using PFL, upper lip thinness and philtrum smoothness but significant difference with additional landmarks concerning the midfacial region	Morphometric analysis of facial landmark data to characterize the facial phenotype associated with FAS
Fang S	2008	South Africa and Finland	5.09 (South Africa) 13.12 (Finland)	86	63	Revised IOM	FAS	3D facial scan, facial feature analysis, feature computation, feature set identification, feature selection	Within the Finnish Caucasian group the automated technique correctly classified 88,2% of the FAS faces and 100% of the control faces; within the Cape Coloured sample 90,9% of FAS faces and 90% of control faces were classified correctly	To develop a computational model that can automatically compute facial features from 3D scans and use this data to identify children with FAS
Mutsvangwa TEM	2009	unclear	Infants/dummies	dummies	5 infants	–	–	Stereophotogrammetry, landmark distances were measured manually and in the software	The stereophotogrammetry precision results were better than those of the manual measurements; the manual measurements showed a higher degree of variability than the stereo-photogrammetry results; concerning stereophotogrammetry, landmarks with with low precision were: the distance from the middle of the philtrum to the crista philtri, pronasale to left subalare, pronasale to right pronasale, philtrum center to labiale superius	Testing of stereophotogrammetric tool for the diagnosis of FAS in infants
Mutsvangwa TEM	2010	Mixed ancestry	FAS 10.90; controls 7.99	17	17	dysmorphologist	FAS	Stereophotogrammetry; 3D coordinates of 26 landmarks, generalized Procrustes analysis, regression and discriminant function analysis were applied to stereophotogrammetrically derived 3D coordinates of landmarks	Analysis showed that the FAS face is characterized by small palpebral fissures, a thin upper lip, and midfacial hypoplasia 5-year old group cassified with 95.46% accuracy and the 12-year old group classified with 80.13% accuracy	Influence of age and size on shape; shape differences between FAS and controls
Jones KL	2010	Native American, Alaskan Native, Asian, Hawaiian/Pacific Islander, Black/African American, White, Cape Coloured, multiracial, unknown ethnicity	Not mentioned	245 FAS	586	Key facial features	FAS	Dysmorphology assessment, additional features	For seven of the eight additional features, there was a „dose-response“ relation with the children in the FAS group having the highest prevalence for each feature	Additional features of FAS, frequency of these features and variability concerning age, sex or race of the child
Astley SJ	2011	Caucasian, African American	6.0–16.9	822	90 6.0–16.9 years + 16 8.3–15.8 years	4-digit code	FASD	Palpebral fissure length, digital face photographs	The mean z-score of the healthy Caucasian group was in accordance with the mean growth curves on the Canadian charts (0.2 SDs) and were 1.6 SDs below the mean on the Hall chart; the mean PFL z-score for children with FAS was 2.4 SDs below the mean on the Canadian charts and 3.9 SDs below the mean on the Hall chart; The charts were not suitable for African Americans	Assess the goodness of two U.S. populations (healthy children & children with prenatal alcohol exposure) when plottet on the Canadian, Hall, and other published PFL charts
Douzgou S	2012	Not defined	5.50	42	?	Reported alcohol exposure	FASD	Genetic testing, e. g. routine karyotype, *in situ* fluorescence hybridisation, Fragile X	Genetic referral did not impact significantly on the general health of the child, the study highlighted that the most useful role of the geneticist was to identify and investigate alternative diagnoses	The study investigated the value of genetic assessment of children with suspected FASD
Suttie M	2013	Cape Coloured (mixed ancestry)		123	69	Hoyme	FAS/pFAS/HE	Dense surface modelling and signature analysis of 3D facial photographs	DSM-based representation of 3D face shape alone achieved perfect agreement for FAS and good agreement for FAS + pFAS; heat map comparison of dynamic morphing of faces to matched controls revealed facial dysmorphism, which was otherwise overlooked	Strategies to help detect facial dysmorphism across the fetal alcohol spectrum in children without classic facial characteristics
Avner M	2014	Not defined	4.8	40	no	Not defined	FASD?	PFL, philtrum smoothness; direct measurement vs. computer software measurement on photographs	Photographic measurements showed shorter PFL lengths than direct measurements; direct measurement scores for philtrum smoothness were different from the computer's measurements using the frontal view but not using the ¾ view	Validation of the computer assisted methods of photographic measurements by comparing the results to those by a trained physician performing direct measurements of the same child
Hoyme HE	2015	Cape Coloured (mixed race)	Not mentioned	400/1,057	no	Not defined	FASD	Two photographic views (frontal/45°); philtrum, vermillion border; Score 1–5 Testing of lip philtrum guide for Caucasian population vs. new lip philtrum guide for Cape Couloured population	New lip philtrum guide for Cape Coloured population; the results indicated more sensitivity of the new guide to detect the cardinal facial features of FASD in a coloured population	Introduction of a lip philtrum guide for a Cape Coloured population
Abell K	2016	South Africa, Italy, United States	5–9	273	981	lip philtrum/Hoyme	FAS/pFAS/ARND	Extraoral mandibular and maxillary arc measurements with a flexible measuring tape, arc ratios	No significant differences in arc ratio measurements, except for males with FAS (ratio increased); female patients with FAS had significantly decreased maxillary arcs	Values for maxillary and mandibula arcs and maxillary to mandibular arc ratio
Suttie M	2017	Cape Coloured/Caucasian	Cape Coloured FAS 10.6/Highliuy Exposes (HE) 10.4; Caucasian FAS 11.8/HE 12.1	Cape Coloured 97 (22 FAS/75 HE)/Caucasian (35 FAS + 73 HE)108	Cape Coloured 69 (mean age10.1)/Caucasian141 (11.5)	Hoyme	FAS/HE	Curvature visualization and quantification; surface curvature-based delineations of the face form	Facial curvature assists the recognition of the effects of prenatal alcohol exposure and helps explain why different facial regions result in inconsistent control-FAS discrimination rates in disparate ethnic groups	Help clinicians detect the facial effects of prenatal alcohol exposure by developing computer-based tools for screening facial form
Valentine M	2017	South Africa, Italy and United States	5–9	89	50	According to May PA, Baete A, Russo J, et al. Prevalence and characteristics of fetal alcohol spectrum disorders. *Pediatrics*. 2014;134 (5):855–866. doi:10.1542/peds.2013–3319	FAS/pFAS/ARND	Manual examination, Dysmorphology scoring system vs. facial dysmorphology novel analysis—computer based recognition, 2D frontal images	Increased diagnostic accuracy for ARND via computer aided method	To compare detection of facial features by computer based recognition software of 2D images against manual examination in FASD
Suttie M	2018	Caucasian/Latin American	13.00	72; (FAS 22; HE 50)	47	Hoyme	FAS/HE	3D image, MRI; facial growth delineations, caudate nucleus and corpus callosum	Combined morphology analysis of the caudate nucleus, and the corpus callosum, with the face better identify patients with FAS; caudate nucleus asymmetry was reduced for patients with FAS	To improve the understanding of the teratogenic effects of prenatal alcohol exposure by simultaneously considering face brain morphology and neurocognitive measures
Blanck-Lubarsch M	2019^a^	Caucasian	8.80 (control group: 8.2)	30	30	4-digit (German FAS Guideline, Landgraf)	FAS	Palatal depth; metric length measurement of the face in vertical dimension; 3D intraoral and facial scans	Palatal depth evaluation did not show significant differences; vertical facial measurements are suitable for FAS diagnosis with midfacial length being significantly shorter and the philtrum length being significantly longer in patients with FAS	To measure intraoral and extraoral dimensions of the maxilla and face in patients with FAS
Blanck-Lubarsch M	2019^b^	Caucasian	8.80 (control group: 8.1)	25	30	4-digit (German FAS Guideline, Landgraf)	FAS	3D facial scans; Philtrum depth measurements	Philtrum depth significantly differs between patients with FAS and healthy controls	To metrically assess philtrum depth in patients with FAS
Blanck-Lubarsch M	2019^c^	Caucasian	8.70 (control group:	28	30	4-digit (German FAS Guideline, Landgraf)	FAS	3D facial scans; inner canthal distance, mouth breadth, palpebral fissure length right/left, distance between alares nasi and sulcus alares nasi	Mouth breadth and breadth between nasal wings cannot be used for FAS diagnosis; nose breadth at the transition point of the sulcus alaris to the philtrum, inner canthal distance (only for males) and PFL are potential indicators for FAS	To investigate metrical difference concerning various facial features in the region of the eyes, nose and mouth
Del Campo M	2021	Not mentioned	1.67–17; mean: 8.33	56 (FAS/pFAS: 16; ARND: 50)	5	Revised IOM	FAS/pFAS/ARND	Telemedicine, face to face examination, 2 examiners	High agreement between telemedecine vs. face to face examination in identification of physical features of FAS	To compare telemedicine and face to face examinations of patients with FASD
Fu Z	2021	Not mentioned	4–18	1,549 facial scans	no	Not mentioned	FASD?	2D images; 14 FAS associated landmarks, regularized transfer learning framework	Results show that the proposed learning framework performs well with limited training samples	Facial anatomical landmark detection using regularized transfer learning with application to fetal alcohol syndrome recognition
Blanck-Lubarsch M	2022	Caucasian	FAS 8.8 Control 8.2	30	30	German FAS diagnostic guideline (Landgraf et al.)	FAS	3D-facial scans, decision trees, support vector machines, k-nearest neighbors, identification of suitable landmarks for FAS	Landmarks with highest scores were midfacial length, palpebral fissure length, nose breadth at sulcus nasi; all tested machine learning methods (k-nearest neighbors, decision trees and support vector machine) showed high accuracy of 89.5%	To identify machine learning methods from 3D facial scans to improve and objectify diagnosis of FAS
Riekman GA	1984	Native American	Not mentioned; primary dentition	19	20	Not mentioned	FAS	Oral hygiene status, dental caries and occlusion, arch length and circumference; full face profile photos, upper and lower dentition photos, teeth in occlusion photos, study models for the control group only study models were utilized	No significant differences were found	Oral findings in patients with FAS
Gir AV	1989	Urban American black chidren	Not mentioned	15	30 (age and ethnicity matched)	Not mentioned	FAS	Lateral cephalograms, 41 linear, angular and projected values were calculated	Forehead is unusually prominent in patients FAS, mandible is of normal overall size, the corpus of the mandible is significantly longer in patients with FAS, overdevelopment of the upper and lower thirds in patients with FAS	To quantitatively assess the craniofacial complex of children with FAS
O’Leary CM	1990	Australian, Aboriginal	Up to 5	84	yes	Prenatal alcohol exposure	FAS	Oral diseases	Children of mothers with alcohol diagnosis had increased odds of gingivitis and periodontal diseases, other diseases of the lip and oral mucosa and diseases of the salivary glands; the odds ratio for children with FAS having any dental admission by 5 years of age was 2.58, the number of children with FAS was too small to examine each of the dental diagnoses separately	Investigate the relationship between maternal alcohol- use disorder and dental hospital admissions in children up to 5 years of age
Rostand A	1990	French	Newborn, first week of life	202	no	Prenatal alcohol exposure	PAE	Standardized morphological examination, 17 craniofacial characteristics, the number of characteristics was calculated for each infant	Offspring of alcoholics and heavy drinkers in the first trimester had significantly more craniofacial characteristics than those of moderate and light drinkers; the authors conclude that craniofacial morphology might be a more sensitive and specific indicator of prenatal alcohol effect than other anomalies	Alcohol use in pregnancy, craniofacial features and fetal growth
Streissguth AP	1991	American Indian, White, Black	12–40	61	no	According to Clarren et al	FAS (70%)/possible fetal alcohol exposed (30%)	IQ according to Wechsler, physical measurements, photographs, medical records, Vineland Adaptive Behaviour scale	Height and head circumference about 2 SDs below the population mean; less effect on weight; characteristic facies of patients with FAS became less distinctive with increasing age; reduced PFL, larger inner canthal distance, abnormalities of the philtrum, lips and misaligned teeth in patients with FAS	Follow- up study of adolescent and adult manifestations of FAS
Church MW	1997	African American, Caucasian	3.8–26.11	22	no	Expert dysmorphologist based on Rosett et al. (1980)	FAS	Hearing assessment, central auditory processing assessment, auditory brain stem response assessment, vestibular system assessment, language and speech assessment	All patients had continuing problems with recurrent serious otitis media throughout childhood; abnormal competing sentence test and abnormal word recognition with ipsilateral noise test; abnormally prolonged auditory brain stem response; normal vestibular function; significant receptive and expressive language deficits below the 5th percentile; dental malocclusions could be found but 52% of the patients had cleft palate	Assessment of patients with FAS for hearing disorders, evaluation of speech, language and vestibular problems, dentofacial anomalies
Moore ES	2002	Caucasian, African American, Other	Under the age of 18	100	31	IOM	FAS (41)/pFAS (59)	Head breadth, minimal fronal breadth, bitragal breadth, bizygomatic breadth, bigonal breadth, interoccular breadth, biocular breadth, PFL, upper facial breadth; midfacial breadth, lower facial breadth, head length, nasal bridge length, nasal length, philtrum length, lower facial height, total facial height, ear length, maxillary arc, mandibular arc, head circumference	19 of 21 measurements differed significantly between FAS, pFAS and control, not significantly different were inner canthal distance and philtrum length; only seven of the measurements differed between pFAS and control, (head length, width, circumference; minimal frontal breadth, bitragal breadth, midfacial depth, total facial height)	Effects of alcohol exposure on the facial phenotype; to objectively describe the anthropometric facial gestalt of individuals diagnosed with FAS and pFAS
Autti-Rämö I	2006	Finnish	Mean 13.00 (8–20 years)	73	No (PFL was compared to American norms Thomas et al. 1987; anthropometric measures to Finnish norms, Sorva et al. 1990)	Revised IOM	FASD	Standard interview regarding family, medical and developmental history; anthropometric measurements, height, weight and head circumference, PFL, morphology of upper lip and philtrum, physical examination to assess major and minor anomalies	The results of the study indicate that the weighted dysmorphology score is a useful adjunctive tool in the clinical and/or research assessment.	To examine a cohort of Finnish children and adolescents with FAS and morphological, societal, cognitive and educational levels to compare the data to those previously published and to examine the utility of a weighted dysmorphology score
May PA	2007	Community in South Africa, other human populations	Different values in table vs. text; Values from table: FAS 10.28; pFAS 6.63; control: 6.29	73 (FAS 55, pFAS 18)	145	Revised IOM	FAS/pFAS	Demographic and growth parameters; dysmorphology score	Height, weight, BMI measures, head circumference, PFL was significantly different between FAS, pFAS and control including total dysmorphology scores, camptodactylie and altered palmar crease were significantly different between the groups	To further explore and summarize the epidemiology, the maternal risk, child characteristics, trends, and etiology of FAS and pFAS
Ervalahti N	2007	Not mentioned	8–16 (mean 11.8)	48 (30 FAS, 13 pFAS, 5 ARND)	No	Revised IOM	FAS/pFAS/ARND	Complete physical examination, dysmorphology score according to Hoyme; cognitive capacity with abbreviated version of the Wechsler intelligence score	Performance on the Wechsler test was below average in all groups; significant difference in total dysmorphology score; more severe growth deficiencies and dysmorphic features were associated with poorer cognitive capacity	To clarify the extent to which dysmorphic features and growth deficiencies are associated with general cognitive capacity in a subsample of the participants
Spohr HL	2007	Caucasian	FAS: 24.7; fetal alcohol exosure (FAE): 21.6	37 (15 FAE; 22 FAS)	no	First examination: Expert in accordance with research society on alcoholism	FAS/FAE	Follow up: dysmorphologist, coded item list based on an interview, the young adult behaviour checklist	Despite substantial reduction of symptoms across time a sizeable number of subject still exhibited growth retardation, microcephaly, developmental delay and hyperactivity. In contrast the very marked craniofacial features mostly disappeared although elongated philtrum and thinner lip were still prominent;only 5% had ever held an ordinary job;young adult behaviour checklist: deviates significant for thought disorder, attention problems, intrusive behaviour, aggressive behaviour.	20-year follow up study
Feldmann HS	2012	White, otherethnicity, unknown	Newborn; 2.6 months	992	no	Alcohol mother, dysmorphologist; Chambers checklist	FASD	Dysmorphologist performed a blinded examination of all infants; patterns of drinking were evaluated by drinks per day, number of binge episodes and maximum number of drinks; timing of exposure was evaluated 0–6 week post conception, 6–12 weeks post conception, 1., 2.,3. trimester	Higher prenatal alcohol exposure in any pattern was significantly associated with the incidence of smooth philtrum but not with short palpebral fissures; strongest associations with timing of exposure were in the second half of the first trimester mostly for thin vermilion border; reduced birth length was increased with exposure in any trimester; the associations were linear and there was no evidence of a threshold	To examine how specific prenatal alcohol exposure patterns relate to characteristic alcohol related facial features and growth deficiencies
Foroud T	2012	South Africa, Cape Coloured (mixed ancestry)	First clinic FAS/pFAS: 5.2; HE: 4.7; control 4.6 Second clinic: FAS/pFAS: 9.20; HE 8.6; control 8.6	76(FAS/pFAS: 35; HE: 41)	49	Revised IOM	FAS/pFAS/HE	3D facial images, 17 anthropometric measurements at 5 and 9 years, IQ; 3 groups (FAS/pFAS, HE and control), prenatal alcohol exposure	Upper facial width, ear length, lower facial depth and eye width were consistent predictors distinguishing those that were exposed from those that were not; prenatal alcohol exposure appears to have its primary effect on brain growth reflected by smaller forehead width; some measures were only predictive at certain ages	To present anthropometric measures that identify and best classify groups of children based on their alcohol exposure and accompanying facial dysmorphology
Nayak R	2012	India	6.21 (cases); 6.16 (controls)	26 (2 FAS; 4 pFAS; 2 neurobehavioural disorder, 18 static encephalopathy)	27 (controls with neurobehavioural disorder, static encephalopathy)	4-digit	FAS	Facial photographic analysis, Waldrop list of minor physical anomalies; child behaviour checklist for 4–16 yrs and 2–3 yrs; Mini international neuropsychiatric interview for children and adolescents; Vineland social maturity scale for below 6 yrs; Malin's intelligence for Indian children	Minor physical anomalies were more common in cases; among FAS facial features, only philtrum smoothness varied significantly; behavioural problems were more pronounced and intellectual functioning significantly poorer in cases	To evaluate the effects among children prenatally exposed to alcohol
Yang Y	2012^a^	Non Hispanic white; Asian; Native Hawaiians; African American; more than one ethnicity; Cape Coloured; European Whites	13.40 (FASD), 13.0 (non-exposed)	69	58	Alcohol mother; Dysmorphologist	FASD	MRI data acquisition, facial and cognitive evaluation	Subjects with FASD showed significantly thicker cortices in several frontal, temporal, and parietal regions; increased inferior frontal thickness correlated with reduced palpebral fissure length	To detect cortical thickness abnormalities in FASD and the association with facial dysmorphology
Yang Y	2012^b^	Non-Hispanic White; Asian, Native Hawaiian; African American; Cape Coloured (mixed ancestry); European Whites	12.80 (FASD); 12.3 (FASD)	82	71	Alcohol mother (maternal interview)	FASD	Facial and cognitive evaluation; MRI data acquisition	FASD subjects had smaller total brain and white matter volumes; FASD subjects consistently showed lower FSIQ (full scale IQ); FASD subjects showed significantly smaller PFL and increased Lipometer scores; significantly reduced callosal thickness in the anterior third and the splenium in FASD; no significant correlations between corpus callosum thickness or area measures and total number of drinks per week or average drinks per occasion in all three trimesters; callosal thickness and area measures were found significantly correlated with facial dysmorphology measures, specifically PFL scores were significantly correlated with callosal thickness (reduced cc thickness had smaller PFLs)	To investigate associations between facial dysmorphology and callosal thickness and area measures
May PA	2016^a^	92.9% Coloured (mixed race); 5.6% Black; 1.5% White	FAS 9.6; pFAS 8.5; ARND 8.7; controls 7.7	341(FAS 173, pFAS 109, ARND 59)	212	Revised IOM	FAS/pFAS/ARND	Height, weight, head circumference; physical growth and dysmorphology score; assessment of cognitive and behavioural traits	Children with FASD were significantly smaller on height, weight, BMI; OFC; PFL, smooth philtrum and narrow vermillion; cognitive and behavioural traits were low, especially in ARND, followed by FAS and pFAS	Prevalence and characteristics of the continuum of diagnosis with FASD were researched in unstudied rural agricultural, lower socioeconomic populations in South Africa
May PA	2016^b^	South Africa	FAS 7.5, pFAS 6.3; ARND 7.7; control 6.3	199 (FAS 69, pFAS 91, ARND 39)	207	Revised IOM	FAS/pFAS/ARND	Cognitive and behavioural traits	Significant differences concerning height, weight, BMI, OFC, PFL, smooth philtrum and narrow vermillion; head significance was a single trait which most distinguished one group from another; minor anomalies: maxillary and mandibular arcs, ICD, IPD; hypoplastic midface, epicanthal folds, ptosis, camptodactyly, flat nasal bridge, altered palmar creases, prognathism; cognitive testing and behaviour checklist low; correlation alcohol use with outcomes	The prevalence and characteristics of the continuum of diagnosis within FASD were researched in a fifth sample in a south African community
Treit S	2016	Caucasian, Aboriginal, other/unknown	12.50 (PAE); 11.9 (controls)	144 (33 FAS/pFAS; 79 ARND or FASD, 32 confirmed prenatal alcohol exposure)	145	Canadian and 4-digit code	PAE/FAS/pFAS	Head circumference and brain volumes from MRI, inter-site reliability; cognitive testing	High inter-site reliability of 0.994 for brain volume and head circumference 0.995; mean head circumference, brain volume and cognitive scores were significantly reduced in the prenatally exposure group, with overlap between groups; no significant correlations between head circumference and any cognitive scores	Relationship between head circumference an brain volume in children with PAE
May PA	2017	Coloured (mixed ethnicity) 92.8%, Black 5.4%; White 1.6%, Other 0.1%	FAS 10.1; pFAS 8.7; ARND 9; controls 10.7	284 (FASD) (FAS 129, pFAS 100, ARND 55)	104	Revised IOM	FAS/pFAS/ARND	Maxillary/mandibular arc; inner canthal distance, interpupillary distance, hypoplastic midface, railroad track ears; ptosis; camptodactyly, altered palmar creases, anteverted nostrils	Shorter innercanthal and interpupillary distance, hypoplastic midface, ptosis, camptodactyly of the fingers, altered palmar creases were significantly more common among FASD groups and most common among children with FAS, even children with ARND have significantly more dysmorphology than controls	Prevalence rates, child characteristics and maternal risk factor in a rural community in South Africa
Blanck-Lubarsch M	2019^d^	Caucasian	8.80 (FAS); 8.2 (controls)	30	30	Specialist pediatrician	FAS	Swallowing pattern, oral habits, breast feeding, speech therapy, ergotherapy, physiotherapy, exfoliation of teeth, DMFT-index, modified DDE index and otitis media were recorded	Swallowing pattern, exfoliation of teeth and otitis media were not significantly different; significant differences could be found concerning mouth breathing, oral habits, age at termination of habits, speech treatment, ergotherapy, physiotherapy and breast feeding; DMFT and DDE-index showed significantly higher values for children with FAS	Tooth malformations, DMFT index, speech impairment and oral habits in children with FAS
Blanck-Lubarsch M	2019^e^	Caucasian	8.70 (FAS), 8.2 (controls)	28	30	4-digit; German Diagnostic Guideline (Landgraf et. al)	FAS	3D-facial scans, 3D-facial parameters at the mouth, nose and eye regions were measured	Significant differences could be found for the distance between left and right sulcus nasi, for the inner canthal distance, as well as for right and left palpebral fissure length; no significant differences for measurements of mouth breadth and breadth between the left and right alares nasi.	To find objective facial parameters for the verification of FAS(D).
Kesmodel US	2019	Denmark	5.20	1,036?; 10 FASD	Not mentioned	4-digit?	FASD, alcohol exposure	Exposure assessment (interview concerning alcohol consumption); digital photos; measurement of three facial features (philtrum, upper lip, lip circularity) on 2D photographs; PFL	Children exposed to 1–4 drinks/week were 8.5 fold more likely to present with FAS/pFAS facial phenotypes, the risk was 2.5 fold increased in children with a single binge exposure in gestational weeks 3–4, the magnitude of expression of the FAS facial phenotype was significantly correlated with all other diagnostic features (growth deficiency, microcephaly and measures of CNS dysfunction)	To use the lifestyle during pregnancy study to assess potential effects of low to moderate weekly alcohol consumption and binge drinking in early pregnancy on facial features associated with FAS
Landgren V	2019	Russia, Poland, Romania, Latvia, Estonia	22	36 (FASD)	No	IOM	FASD	Clinical evaluation of social, medical, psychiatric, neuropsychological, adaptive and ophtamological status by a physician ophthalmologist, orthoptist and psychologist	Follow-up study of 15.5 years; in 21 individuals with FAS growth restriction in height and head circumference of approximately 1.8 SD persisted into adulthood; 56% were dependent on social support; 69 had gross motor coordination abnormalities; ophtamological abnormalities in 29/30; median IQ of 86 in childhood had declined significantly to 71 in adulthood; psychiatric disorders in 88%, most commonly attention deficit hyperactivity disorder; BMI steadily increased from start to adulthood; the proportion exhibiting small palpebral fissures had decreased considerably by adulthood (76% vs. 47%)	To characterize young adult outcome of FASD in a socially favourable rearing environment and to evaluate the diagnostic stability of FASD in the adulthood
Gomez DA	2020	Cape Coloured (mixed ethnicity)	6.93	1,395; FAS 582; pFAS 481; ARND 332	604	Revised IOM	FAS/pFAS/ARND	Plastic ruler; ICD, IPD; OCD	Reductions in all three centiles ICD, IPD and OCD could be observed	We investigated how inner canthal distance, inter pupillary distance and outer canthal distance centiles differed between FASD and non-FASD individuals
Da Silva K	2021	Not mentioned	FASD 8.37; control 8.34	68	184	Guideline not mentioned, information from medical intake form, medical records	FASD	Retrospective study, oral health status	Children with FASD were significantly older at their first dental visit, had significantly higher DMFT/dmft. (score: 7.18 vs. 2.93); children with FASD were 4.71 times more likely to be referred for treatment under general anaesthesia	To investigate differences in oral health status indicators, treatment outcomes and risk factors for treatment under general anaesthesia for children with FASD compared to healthy controls
Jacobson SW	2021	Cape Coloured (mixed ancestry)	Early childhood (2005): FAS 6.5; pFAS 6.4; highly exposed 4.6; controls 5.1 School age (2009;+4 years) Puberty (2013;+4 years) Adolescence (2016;+3 years)	96; FAS 18; pFAS 27; highly exposed 51	59	Revised IOM	FAS/pFAS	Evaluation by dysmorphologists; weighted dysmorphology according to Hoyme	Prevalence of the physical phenotype was stable across the four ages for about half of the children with FAS and about one third of those with pFAS; the physical phenotype was most apparent during early childhood and least apparent during puberty, due to differences in timing of the growth spurt and evolving adult face; short palpebral features and small head circumference diminished with age, flat philtrum fluctuated while thin vermillion, weight and height restriction were stable	Findings from longitudinal study on the evolution of the physical phenotype of FAS and pFAS from early childhood to adolescence
May PA	2021	Cape Coloured (mixed ethnicity) 68%; black African 16%; White 15%; Other 1%	7.2 (FAS); 7.1 (pFAS); 7.1 (ARND); 6.9 (controls)	181 FASD; (48 FAS, pFAS 65; ARND 67; ARBD 1)	137	Revised IOM	FAS/pFAS/ARND/ARBD	2D photographs; dysmorphology examination; mother interview; cognitive and behavioural traits; OFC; BMI; height; weight; PFL; smooth philtrum; narrow vermillion of upper lip	Children with FAS, pFAS, ARND were significantly different from controls on all cardinal variables, multiple dysmorphology traits and neurobehavioural performance; women reporting first trimester drinking of 2 drinks per drinking day were 13 times more likely to having a child with FASD and those who reported drinking throughout pregnancy were 19.4 times more likely to having a child with FASD	Analysis of the common physical and neurobehavioural traits; association between alcohol and physical features

Some studies only consulted expert dysmorphologists/pediatricians for FASD diagnosis (14 studies) or did not state which guideline was used (10 studies). For diagnosis, eight studies only considered the amount of alcohol consumed by pregnant women during pregnancy. 4 studies mentioned Hoyme as reference, two studies performed the Gestalt method. One study used the Chambers checklist, one study mentioned May et al. as reference and 1 study mentioned Clarren et al. as reference.

### 3.7. Age groups

The majority of studies only included infants or school children. Only three studies included adult patients. Three studies did not mention the patients' ages. 27 studies included children aged between 6 and 10 years, 10 studies included children 5 years or younger, 7 studies included children 10–14 years old. Only two studies included patients aged above 18 years. Some studies had a large age range (each 1 study: 0–27 years, 6–17 years, 1–17 years, 4–18 years, 12–40 years, 3–26 years, 8–16 years).

### 3.8. Group sizes

For FASD patients 7 studies included 20 subjects or less, 11 studies included 35 patients or less, 6 studies included 50 subjects or less, 16 studies included 100 patients or less and 19 studies included more than 100 participants.

For patients with FAS 10 studies included 20 patients or less, 14 studies included 35 patients or less, 4 studies included 50 subjects or less, 13 studies included 100 participants or less and 5 studies included more than 100 participants.

### 3.9. Ethnicities

Ten studies did not mention which ethnic group was analyzed. Many studies analyzed different ethnic populations. Some of these studies separately measured and analyzed these groups while other studies did not differentiate between different ethnicities.

### 3.10. Methods

Methods included direct facial measurements [7 studies (38–44)], 2D-photos [13 studies (20, 35, 45–52, 53–55)], 3D photographs [6 studies (27, 29, 33, 34, 56, 57)] and 3D-scans/stereophotogrammetry [9 studies (21–25, 28, 31, 32, 58)], landmark measurements, cephalometric measurements [2 studies (59, 60)], oral status [4 studies (61–64)], demographic and growth parameters [1 study (65)], heat maps [1 study (33)], telemedicine [1 study (66)], machine learning [1 study (21)] or automated diagnosis [7 studies (27, 31, 35, 50, 56, 58, 67)]. For decision trees the landmarks with highest scores for FAS detection in one study were midfacial length, palpebral fissure length and nose breadth at sulcus nasi (21).

### 3.11. Orofacial parameters

Small palpebral fissure length (PFL), smooth philtrum, upper lip circularity, thin vermillion border, midfacial hypoplasia, small head circumference and high dmft/DMFT (decayed, missing, filled teeth) score were the parameters, which were frequently found in the orofacial region.

#### 3.11.1. Facial characteristics

##### Eyes

Reduced palpebral fissure length was found in 19 studies (20, 21, 23, 27, 29, 31, 38, 45, 46–48, 55, 57, 63, 65, 71). One study mentioned that higher alcohol exposure was not correlated with smaller PFLs and one study found that reduced PFLs were less frequently found in adult patients with FASD (42, 71). 4 studies found reduced IPD or ICD (43, 44, 63, 70). Epicanthal folds and ptosis were described by one study (70).

##### Lips and philtrum

12 studies (20, 24, 30, 38, 41, 42, 47, 49, 51, 55, 69, 70) found a smooth philtrum in patients with FASD and 12 studies (18, 30, 32, 38, 41, 47, 55, 68) reported a thin vermillion border or reduced upper lip thickness. Philtrum depth, when measured metrically on 3D-facial scans, significantly differed between patients with FAS and healthy controls (24).

##### Nose

One study found that nose breadth at the transition point of the sulcus alaris to the philtrum could be an indicator for FAS (23). Another study describes a flat nasal bridge (70).

##### Facial proportions

A hypoplastic or flat midface was mentioned in four studies (32, 43, 45, 70). Midfacial length was found to be significantly shorter when using profile analysis (22). Another study found overdevelopment of the upper and lower thirds in patients with FAS (60).

##### Impact of age on facial parameters

One study describes a decreased proportion of patients exhibiting small palpebral fissures in adulthood (73). Another study also describes fading of short palpebral fissures, whereas a thin vermillion was still apparent and the flat philtrum was variable. This study also describes that the physical phenotype of FAS was most apparent during early childhood and least apparent during puberty (71). Another study describes fading of facial parameters with age with the exception of elongated philtrum and thinner lips (72).

##### Impact of alcohol on facial findings

One study found that the offspring of alcoholics and heavy drinkers in the first trimester had significantly more craniofacial characteristics than those of moderate and light drinkers (40). Another study describes that higher prenatal alcohol exposure in any pattern was significantly associated with the incidence of a smooth philtrum but not with short palpebral fissures (42). In one study, children exposed to 1–4 drinks/week were 8.5-fold more likely to present with FAS phenotypes and the risk was 2.5-fold increased in children with a single binge exposure in gestational week 3–4 (52).

#### 3.11.2. Findings of the dentition

Three studies found significantly higher dmft/DMFT scores in patients with FASD (1 of these studies could only find higher dmft and not DMFT scores) (61, 63, 64). One study found that the DDE-index measuring structural tooth anomalies was significantly higher in patients with FAS (63). Another study describes misaligned teeth (55).

#### 3.11.3. Findings of the upper and lower jaw bones

Differences in mandibular and maxillary arcs were found in two studies (39, 43), abnormal vertical facial measurements, such as vertically and horizontally underdeveloped maxilla, large gonial angle and a short ramus, tendency to anterior open bite were found in three studies (22, 61, 74). One study found that the mandible was of normal overall size whereas the corpus of the mandible was significantly longer in patients with FAS (60). Another study described prognathism (43).

#### 3.11.4. General oral health

Two studies found increased odds for gingivitis or periodontal diseases (60, 62). The odds ratio for children with FAS of having any dental admission by 5 years of age was 2.58 (62). Patients with FASD were 4.71 times more likely to be referred for treatment under general anaesthesia in one study (64). Additionally, one study found significantly higher rates of mouth breathing and habits in patients with FASD (63).

## 4. Discussion

This review shows that various parameters in the orofacial region for the diagnosis for FASD have been found so far. However different diagnostic methods or differing landmarks (for example manual/automated vs. 2D/3D measurements) for the same characteristic were used, which leads to difficulties in matching study results into uniform criteria.

In parts this is due to a multitude of heterogeneous diagnostic guidelines, which exist for the diagnosis of FASD. These diagnostic guidelines have in parts similarities but are not comparable or universal (12, 13). Riley et al. compared four different guidelines and could show that they are comparable in the sense that they all use facial characteristics, growth retardation, CNS involvement and alcohol exposure as the cardinal features (13). However, different cut-off values for diagnosis are used, for example the four-digit code requires a head circumference which is smaller by more than two standard deviations below the 2.5th percentile, whereas the revised IOM and the CDC require only measurements below the 10th percentile for FAS diagnosis.

The studies analyzed in this review most often used the IOM or revised IOM criteria, as well as the 4-digit diagnostic code or the German diagnostic guideline (5, 6, 14, 36, 37). Many studies however, only stated the verification of FASD via diagnosis by an expert dysmorphologist or reported alcohol consumption of the mother during pregnancy.

This lack in comparability and diagnostic standard concerning verification of a FASD diagnosis shows the importance of further clarification and standardization of this process. Another difficulty is that most studies included different ethnicities and therefore are not comparable. In addition, values or parameters for adults are not available. Only three studies investigated facial features in adult patients and Streissguth et al. concluded that characteristic facial features became less distinctive with increasing age (55, 72, 73).

Much progress seems to have been made concerning the investigation of new, objective and innovative diagnostic methods such as 3D-facial scans, stereophotogrammetry and machine learning approaches, which could facilitate and improve the diagnostic process in the future (20–35).

Many studies used 2D photographs in combination with the lip-philtrum guide as well as ruler measurements for the palpebral fissure length. Even if these are well-established methods in FASD detection, it seems that 3D measurements may be more accurate. Many studies used 3D-facial scans or 3D-photographs for further landmark analysis (21–25, 27, 29, 30, 32–34, 56–58).

Astley et al. compared accuracy of measurements of fissure length with a ruler with using the FAS facial photographic analysis software and found that the software was more accurate in comparison with a handheld ruler, which showed high interrater variability (75).

Meintjes et al. found high repeatability of PFL, ICD and IPD for 3D measurements in comparison with measurements with a handheld ruler (29).

A study by Mutsvangwa et al. could show that stereophotogrammetry precision results were better than those of manual measurements (31). Fang et al. found high precision for correct classification of FAS faces via automated techniques (59).

Suttie et al. found that facial curvature method assists the recognition of the effects of prenatal alcohol exposure (67). Another study by Suttie et al. could show that dense surface modelling achieved good agreement for FAS and pFAS and that heat map comparison of faces to matched controls revealed facial dysmorphisms with were otherwise overlooked (33). Valentine et al. also found an increased diagnostic accuracy for ARND via computer aided methods (35).

Machine learning methods such as decision trees, support vector machines and k-nearest neighbors proved to be accurate methods in detecting certain facial features in a study by Blanck-Lubarsch et al. (21). The above study results support that research is needed towards using 3D measurements as well as computer aided analysis methods or machine learning techniques since these methods seem to be accurate for FASD diagnosis.

Concerning the FASD phenotype, there were some parameters, which were repeatedly measured and mentioned as diagnostic features in FASD. These parameters comprise reduced palpebral fissure length, smooth philtrum, thin vermillion border and upper circularity, abnormal interpupillary or innercanthal distance and hypoplastic or flat midface (22, 23, 46, 48, 49, 70).

Unfortunately, the measuring or detection methods for most parameters were not metrically comparable between different studies because of different methodology (for 2D vs. 3D measurements) so that it was not possible to extract definite values for certain FASD parameters. For future research standardized measuring would improve comparability and enable the documentation of norm values for FASD diagnosis.

To date palpebral fissure length, smooth philtrum and thin vermillion border are cardinal parameters for the diagnostics of facial parameters in FASD diagnosis of most FASD guidelines (5, 6, 14, 15).

However, studies found that not all parameters are detectable in adulthood and another difficulty is that comparing facial parameters of patients to photographs in the lip-philtrum guide needs an expert dysmorphologist in the field of FASD (71–73).

Study results in various studies showed that multiple facial characteristics such as interpupillary or innercanthal distances, vertical measurements in x-rays or 3D-photographs, as well as nasal, maxillary or mandibular measurements could be used for FASD diagnosis (5, 6, 14, 15, 18, 21, 67, 74).

In addition, patients with FASD seem to have more dental and orthodontic treatment needs in comparison with healthy controls. Studies found higher dmft/DMFT values and more anomalies of the enamel structure (DDE-index) for FASD patients. Orthodontic findings included anterior open bite, horizontally and vertically underdeveloped maxilla, as well as prognathism. Mouth breathing and higher rates of habits were also found and can contribute to orthodontic problems such as open-bites, crossbites or increased sagittal distances between upper and lower front teeth (76). One study found a higher prevalence of crossbites and dental crowding in patients with FASD. These orthodontic problems need early treatment since dental crowding tends to aggravate with age and crossbites lead to asymmetric growth of the mandible or growth restriction of the upper jaw (77–83). Therefore, these dental and orthodontic parameters could not only contribute to the detection of FASD but also need to be kept in mind as these findings show the necessity for interdisciplinary treatment and early referral of the FASD patients to the dentist and/or orthodontist for prevention (43, 55, 61, 63, 64, 83). The included studies did not investigate dental findings in the deciduous dentition apart from dmft scores. In addition, it might be interesting to investigate, whether dental findings concerning crossbites or dental crowding are still characteristic parameters in the permanent dentition or whether these factors might be less frequent in adults because of orthodontic treatment in adolescence. Dental crowding is a malocclusion, which becomes worse with age even in healthy subjects (78), which in turn could result in dental crowding not being a distinguishing characteristic for FASD in adults. Therefore, these orofacial findings should be investigated for different age groups.

Further research should aim at finding values for abnormal parameters in FASD and defining universal, favorably digital measuring methods. The latter would help to store the data more easily and provide it to bio databanks, that should be accessible worldwide.

Once homogeneous and easy to apply measuring methods for the detection of FASD are defined, a routine screening of small patients could be applied by pediatricians within routine check-ups, or to for example kindergarten or primary school children, thus enabling early developmental care for patients with FASD. Early detection of FASD seems even more important since studies found a possible fading of facial parameters with age and in adult patients thus making diagnosis at older ages more difficult (55, 71, 73). If the applied methods were universal across different countries, this would allow for comparability and improvement of the diagnostic process. In addition, routine screening procedures in early childhood could reduce the number of undetected FASD patients which is suspected to be high across the world.

Furthermore, finding additional facial parameters could improve the development of machine learning methods in FASD screening.

Further studies investigating facial features in adults could find helpful parameters for the diagnosis of patients with FASD, which were not diagnosed in childhood or adolescence.

The results of this review stress the need for uniform diagnostic criteria and measuring methods. Comparable values are necessary in order to create bio databases with values for different degrees, ethnicities and ages.

## 5. Conclusion

This review could show that uniform diagnostic criteria and parameters for the orofacial region in FASD diagnosis are needed. Many facial parameters in the orofacial region for the detection of FASD have been found so far but only few of them are part of the diagnostic processes or current guidelines.

Further research should aim:

- to find among the existing characteristics the most comparable parameters
- to find parameters which are easy to measure for routine pediatric screenings as it is important to detect patients at young ages, even more since there seems to be a fading in facial parameters with age
- to examine larger groups of different ethnicities to find objective and uniform criteria
- to create a bio database with values and parameters for different ethnicities, degrees and age groups
- to address machine learning and 3D-facial measurement approaches as these seem to be more accurate and might facilitate the diagnostic process
- to find a consensus for one globally valid guideline with special consideration of different ethnicities and age groups

In addition, several orofacial findings hint at a higher need for dental and orthodontic treatment. This stresses the need for early referral to a dentist or orthodontist.
